# VEGF Concentration in a Preovulatory Leading Follicle Relates to Ovarian Reserve and Oocyte Maturation during Ovarian Stimulation with GnRH Antagonist Protocol in In Vitro Fertilization Cycle

**DOI:** 10.3390/jcm10215032

**Published:** 2021-10-28

**Authors:** Wen-Bin Wu, Hsuan-Ting Chen, Jun-Jie Lin, Tsung-Hsuan Lai

**Affiliations:** 1 School of Medicine, Fu-Jen Catholic University, No. 510, Zhongzheng Rd., Xinzhuang Dist., New Taipei City 242062, Taiwan; 059299@mail.fju.edu.tw; 2 Graduate Institute of Biomedical and Pharmaceutical Science, Fu-Jen Catholic University, No. 510, Zhongzheng Rd., Xinzhuang Dist., New Taipei City 242062, Taiwan; 3 Ph.D. Program in Pharmaceutic Biotechnology, Fu-Jen Catholic University, No. 510, Zhongzheng Rd., Xinzhuang Dist., New Taipei City 242062, Taiwan; jenny123142003@gmail.com; 4 Department of Obstetrics and Gynecology, Cathay General Hospital, No. 280, Renai Rd. Daan Dist., Taipei 10693, Taiwan; junjin.bow@gmail.com

**Keywords:** angiogenesis, folliculogenesis, IVF, ovarian reserve, oocyte maturation, VEGF

## Abstract

Serum vascular endothelial growth factor (VEGF) is involved in follicular vascularization, oxygenation, and consequently in oocyte maturation and embryo development. Unanswered questions remain regarding the relationship of intrafollicular VEGF level in preovulatory leading follicles to oocyte maturation and ovarian reserve during ovarian stimulation. We conducted this study to investigate the relationship of intrafollicular VEGF level in the fluid of single preovulatory leading follicles to ovarian reserve and oocyte maturation in patients receiving GnRH antagonist in vitro fertilization (IVF) protocol treatment. One hundred and eighty-five patients receiving IVF treatment were recruited and assigned to low-, normal-, and high-ovarian-reserve groups according to their serum anti-Müllerian hormone (AMH) level. Follicular fluid (FF) in preovulatory leading follicles, serum profiles, and clinical variables were collected for analysis. The result disclosed a significant among-group difference in FF VEGF concentration. Moreover, the serum AMH level was also negatively correlated with FF VEGF level. The oocyte maturation rate tended to be increased at higher AMH levels. FF VEGF concentration was significantly positively correlated with basal FSH level. In conclusion, FF VEGF concentration has a negative association with ovarian reserve level and oocyte maturation rate in patients undergoing GnRH antagonist IVF protocols.

## 1. Introduction

Follicle growth in humans involves multiple events that alter the follicular microenvironment required for oocyte development. In general, well-conditioned follicles are highly vascularized; nevertheless, vascularity in atresia follicles is usually poor, implying the existence of an association between follicular vascularization and follicle development [1]. With Doppler ultra-sonographic evaluation, Huey’s team discovered that oocytes derived from well-vascularized follicles and well supplied with oxygen had better fertilization and developmental competence [2]. High-grade vascularity associated with better in vitro fertilization (IVF) outcomes had been observed in transvaginal Doppler ultrasound studies of perifollicular vascularity [3]. Therefore, angiogenesis plays a key role in follicle growth and IVF outcomes.

In folliculogenesis, angiogenesis is a complicated process that involves numerous chemokines/cytokines in the ovary. These dominant paracrine factors are vascular endothelial growth factor (VEGF), insulin-like growth factor (IGF), angiopoietin (ANPT), fibroblast growth factor (FGF), hypoxia-inducible factor (HIF), and bone morphogenetic proteins (BMPs) [4,5]. Among these factors, VEGF is the major player of follicular angiogenesis [6]. In many species, VEGF is discovered in follicular granulosa cells (GCs) and theca cells (TCs). It is a key factor affecting the development of the vascular network surrounding the theca layer [7,8] and the main inducer of vessel hyperpermeability and endothelial cell proliferation and migration [9]. Previous reports showed that VEGF is detected in the GCs, TCs, and follicular fluid (FF) of the primate ovary [10,11,12]. VEGF also functions in follicular angiogenesis and intrafollicular oxygenation, subsequently affecting follicular maturation, oocyte quality, fertilization, and the developmental potential of embryos [13,14,15].

The factors inducing VEGF secretion during folliculogenesis are not fully understood in IVF cycles. It is obvious that intrafollicular and perifollicular factors could collaboratively stimulate VEGF secretion. Follicule-stimulating hormone (FSH) is reported to induce VEGF secretion in GCs [16]. In addition, transforming growth factor (TGF)-β1 and FSH are reported to increase the secretion of VEGF, resulting in enhanced angiogenic activity in the rat ovary [17]. The luteinizing hormone (LH) concentration is also related FF VEGF concentration. It has been suggested that VEGF from GCs in pre-ovulatory follicles, induced by LH surge, is a mediator of luteinization in women with natural menstrual cycles [18]. The stimulation of GCs by human chorionic gonadotropin (hCG), IGFs, and HIF can upregulate the expression of VEGF, which can impact the remodeling of vascular endothelial cells and the formation of the corpus luteum [19,20].

In the past, many tests were developed to predict the outcomes of IVF. Some important measures of oocyte quality and quantity are used to predict ovarian response and pregnancy rate, including serum basal FSH level, estradiol level, antral follicle count (AFC), anti-Müllerian hormone (AMH) level, and retrieved oocyte number. However, the accuracy of IVF outcome prediction cannot be totally supported [21]. Basal FSH level is one of the most commonly used indicators for predicting IVF outcome. Women who have higher basal FSH levels may have lower ovarian reserve and respond poorly to gonadotropin stimulation. Subsequently, they exhibit a lower pregnancy rate and live-birth rate [22]. Serum AMH level is suggested as a better predictor than basal FSH, estradiol, AFC, diminished ovarian reserve, and the possibility of pregnancy in natural and assisted reproduction [23,24]. Serum AMH less than 0.7 ng/mL indicates poor ovarian reserves and significantly lower pregnancy rates in the general population [25]. Therefore, diminished or poor ovarian reserves are associated with low response to ovarian stimulation and lower pregnancy rates in IVF cycles.

Ovarian reserve decreases with age and is often linked to oocyte quantity and quality [26]. Notably, however, oocyte quantity can also be used as an independent predictor of oocyte quality and live-birth rate. Some reports showed that women with higher numbers of retrieved oocytes have a higher pregnancy rate and live-birth rate [27,28,29]. It has also been shown that oocyte quantity and oocyte quality have a significant impact on the cumulative live-birth rate [30] and that oocyte number and number of top/good-quality embryos are positively correlated [31].

To date, the regulation of intrafollicular angiogenesis and the clinical factors affecting regulation have not been well established in human IVF cycles. The question remains as to whether intrafollicular VEGF concentration in a preovulatory leading follicle is associated with oocyte maturation and ovarian reserve, and what clinical factors are related to the secretion of VEGF in FF during ovarian stimulation with a GnRH antagonist. Thus, the aim of this study was to investigate the intrafollicular VEGF level in individual preovulatory leading follicles and its association with ovarian reserve level and oocyte maturation in infertile women undergoing IVF GnRH antagonist protocols.

## 2. Materials and Methods

### 2.1. Patient Selection and Grouping

The present study was approved by the Institutional Review Board of Cathay General Hospital, Taipei, Taiwan. All participants signed an informed-consent form before enrollment (IRB No.: CGH-FJ105002).

We included candidates with 18.0 ≤ BMI ≤ 30.0 kg/m^2^, with age <45 years, in the first IVF cycle, using a GnRH antagonist protocol, and with basal FSH <15 mIU/mL. The criteria of exclusion were the presence of ovarian benign pathologies, ovarian cancers, repeated IVF treatment, non-GnRH antagonist protocols, premature menopause, and sexually transmitted diseases. Patients with BMI < 18.0 kg/m^2^ or BMI > 30.0 kg/m^2^, and male infertility factor were also excluded from the study.

A total of 185 IVF patients were enrolled between August 2016 and July 2017 and assigned to three groups according to AMH level: 81 patients with AMH < 2 ng/mL (Group A), 81 patients with AMH between 2 ng/mL and 6.8 ng/mL (Group B), and 23 patients with AMH > 6.8 ng/mL (Group C).

### 2.2. Protocol Procedures

#### 2.2.1. Ovarian Stimulation

A GnRH antagonist protocol was used for ovarian stimulation. When the IVF treatment started, gonadotropins with recombinant FSH (Gonal-F, Merck Serono, Darmstadt, Germany) plus highly purified urinary hMG (Menopur, Ferring, Gentilly, France) were administered on cycle days 2 or 3 at different doses depending on AMH level, age, basal FSH level, body weight, and AFC. The dose was adjusted during ovarian stimulation based on the ovarian response to the starting dose. Pituitary suppression at the late follicular phase was initiated by daily injection of 0.25 mg of cetrorelix acetate (Cetrotide, Merck Serono) when follicles exceeded 14 mm in diameter or when serum estrogen levels (E2) were in excess of 500 pg/mL. When at least 3 follicles were more than 18 mm in diameter, ovulation was triggered by dual injections of 3000 U of hCG (Ovidrel, Merck Serono) and 0.2 mg of GnRH agonist (Decapeptyl, MSD, Merck). Oocyte retrieval via the guidance of transvaginal ultrasound was performed at 35–37 h after the dual trigger.

#### 2.2.2. Follicular Fluid (FF) Samples and Oocyte Retrieval

We analyzed an FF sample from *a preovulatory leading follicle* (18–22 mm in diameter) during oocyte retrieval instead of pooling multiple FF samples (from non-leading and leading follicles) in one container, which made the results more precise when evaluating the association of intrafollicular VEGF level with oocyte maturation and ovarian reserve level. The FF was aspirated under transvaginal-ultrasound guidance as previously described [32,33]. Numerous oocytes were obtained from the preovulatory leading follicles (18–22 mm in diameter) in the ovary of each IVF patient, but only one FF sample was collected from the first retrieving single preovulatory leading follicle. Therefore, for each IVF patient, only one tube was used for FF aspiration and only one FF sample was collected from one preovulatory leading follicle of an IVF patient. The FF collection procedure was proceeded very carefully to avoid blood contamination, and the FF was obtained without flushing by culture medium, minimizing the wash medium left in the tube that would dilute the FF during collecting. If blood contamination occurred, that FF sample was discarded. Otherwise, the obtained FF was then further centrifuged at 1000× *g* for 3 min to remove possibly contaminated blood cells or cell debris. The collected FF sample was aliquoted to the tubes and stored at −80 °C for further analysis.

#### 2.2.3. Measurement of Intrafollicular VEGF Level

The intrafollicular VEGF concentration was measured by using a Human VEGF ELISA Development kit (R&D Systems, Inc., Minneapolis, MN, USA) according to the manufacturer’s protocol. The details of the method were described in our previous study [32].

#### 2.2.4. Serum Hormone Assays

Blood tests were performed on cycle day 2 and the hCG trigger day. The concentrations of serum AMH in all subjects were measured before the start of IVF treatment. The concentrations of FSH, LH, E2, and progesterone were quantitatively determined by chemiluminescence assay (Abbott Biologicals B.V., Olst, Overijssel, The Netherlands) as described in our previous work [32]. We defined the detection limit as follows: FSH = 0.06 mIU/mL, LH = 0.09 mIU/mL, E2 = 10 pg/mL, and P = 0.1 ng/mL. For AMH measurement, all samples were immediately assayed with a commercial ELISA kit (AMH Gen II assay, Beckman Coulter, Brea, CA) according to the manufacturer’s instructions. The intra- and inter-assay coefficients of variation did not exceed 6% and 16%, respectively.

### 2.3. Statistical Analysis

The data are expressed as the mean ± standard deviation (SD). Unpaired Student’s *t-*tests were performed to compare the means of two groups. One-way analysis of variance with multiple comparisons was used to test the differences in the means between groups. The degree of correlation between parametric variables was measured by using the Pearson’s correlation coefficient, and Spearman’s ρ was used for nonparametric variables. A value of *p* < 0.05 was considered statistically significant. All data were analyzed with a commercial statistical package SPSS, version 16.0 (SPSS, Chicago, IL, USA).

## 3. Results

### Main Results

A total of 185 patients were included in the study. Baseline characteristics and laboratory measurements of the groups of patients are compared in Table 1. There were no significant differences among the three groups in terms of BMI, basal LH level, basal E2 level, basal P4 level, total FSH dose, total LH dose, P4 level on hCG day, and the rate of oocyte maturation. However, there were significant differences among the groups in age, AMH level, AFC level, basal FSH level, stimulation duration, E2 and LH levels on hCG day, intrafollicular VEGF concentration, oocyte number, and the number of mature (MII) oocytes (*p* < 0.05) (Table 1). The ovarian response increased in association with AMH and AFC levels and decreased with age and basal FSH level.

**Figure 1:**
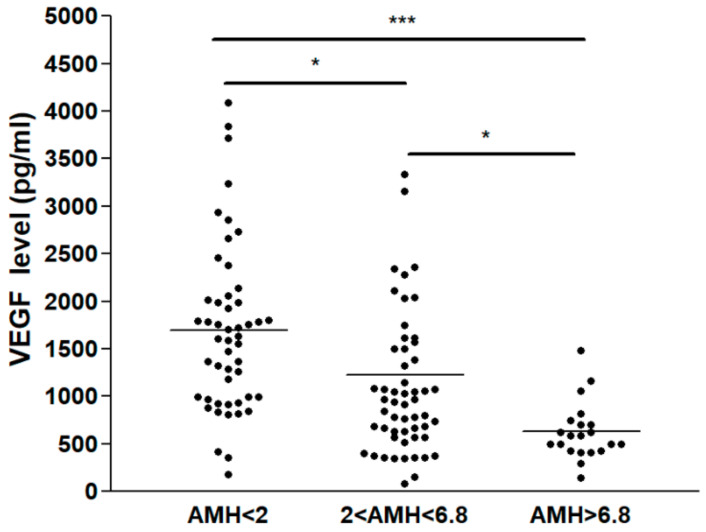
Relationship of VEGF concentration in follicular fluid (FF VEGF concentration) to AMH level. FF VEGF concentration in different AMH groups of IVF patients. Statistical difference: * *p* < 0.05 and *** *p* < 0.01 between the AMH groups.

**Figure 2:**
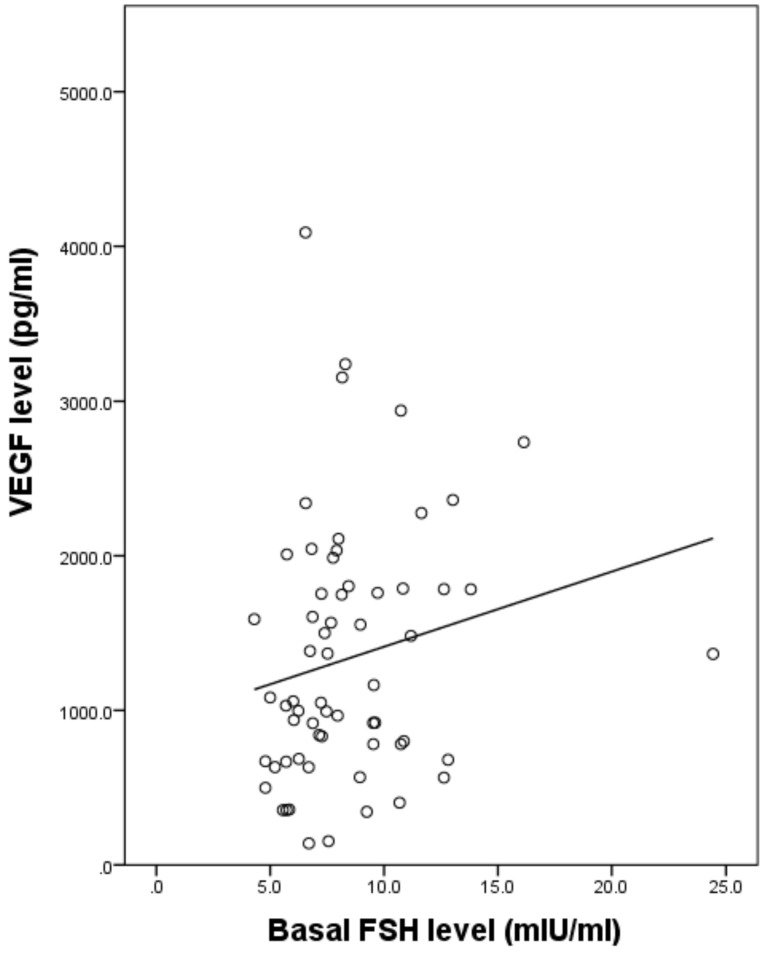
Correlation between intrafollicular VEGF level and basal FSH level. The correlation between intrafollicular VEGF and serum basal FSH level was weakly positive (Spearman coefficient = 0.274, *p* < 0.05).

**Figure 3:**
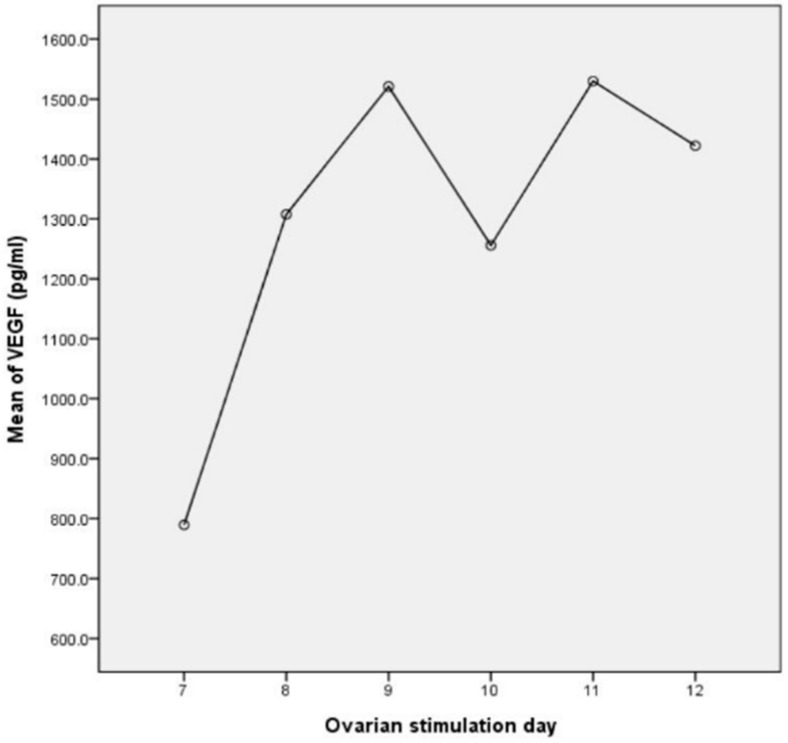
Correlation between intrafollicular VEGF level and number of ovarian-stimulation days. Intrafollicular VEGF secretion was increased with the number of days of ovarian stimulation. The secretion level reached a plateau on stimulation day 9 but then dropped transiently on stimulation day 10.

Overall, there were significant differences in VEGF concentrations among the three groups (*p* < 0.05). Interestingly, VEGF concentration in FF varied between 138.9 and 4089.5 pg/mL. The mean VEGF level was 1424.1 ± 146.9 pg/mL. At this level, which is within the ng/mL range, VEGF was highly suspected of being physiologically and functionally active in vivo. VEGF levels in FF were higher in Group A (1692.2 ± 124.1 pg/mL) than Group B (1222.4 ± 137.8 pg/mL) and Group C (627.9 ± 66.7 pg/mL) (Table 1). Moreover, there was a negative association between serum AMH level and FF VEGF concentration in all three groups (Figure 1). In parallel, the oocyte maturation rate was increased when serum AMH level was higher, i.e., it was lower in Group A (66.3%) than in Group B (72.2%) and Group C (75.7%), and the differences between groups (A vs. B and A vs. C) reached statistical significance (*p* < 0.05) (Table 1). Next, the association of FF VEGF concentration and serum hormone level or some other measurements was investigated. The FF VEGF concentration had a weak positive correlation with basal FSH level (Spearman coefficient = 0.274, *p* < 0.05) (Figure 2), but it was not associated with the serum E2 level on the hCG day and gonadotropin dose. Remarkably, VEGF secretion in FF increased with the number of ovarian-stimulation days. The secretion was maximal and reached a plateau on stimulation day 9 (Figure 3).

## 4. Discussion

As reported in the previous studies with GnRH agonist long protocol, a higher VEGF level in FF is involved in impaired folliculogenesis via poor angiogenesis due to hypoxia in the ovary [6,34,35,36,37]. Previous studies reported that aging women have increased levels of VEGF in the FF [38,39,40]. Elevation of oxidative stress results in an increase in VEGF level [41]. When local hypoxia occurs, VEGF mRNA will be upregulated [42]. The presence of elevated VEGF levels combined with decreased blood flow around the follicle indicates that this could result from possibly defective signaling pathways or an increased gap between the perifollicular layer and the wall of the growing follicle in relation to aging [43]. However, these studies were obtained by analyzing multiple FF from immature to mature follicles. Therefore, in this study, we analyzed the intrafollicular VEGF concentration from a single preovulatory leading follicle, which made the results more precise on the association of intrafollicular VEGF level with oocyte maturation and ovarian reserve. We also observed that higher basal FSH levels are associated with higher intrafollicular VEGF levels (Figure 2). Overall, we found that low ovarian reserve is related to a higher VEGF concentration in FF during ovarian stimulation. In addition, low ovarian reserve and high basal FSH level could increase intrafollicular VEGF concentration in a preovulatory leading follicle. This implies that intrafollicular hypoxia causes VEGF secretion to increase, even in the preovulatory leading follicles of the patients with low ovarian reserve.

Healthy follicles are highly vascularized, whereas those undergoing atresia have poor vascularity [1]. Moreover, angiogenesis plays a key role in folliculogenesis, in which VEGF is one of the key angiogenic factors [44]. Therefore, the possible explanations for low ovarian reserve related to a higher VEGF concentration in FF during ovarian stimulation may be summarized as follows. Patients with low ovarian reserve may have more follicular angiogenesis to promote vascularization, while patients with higher ovarian reserve may have less follicular angiogenesis. Moreover, the action mechanism involved in intrafollicular VEGF induction may result from follicular hypoxia, as previously described by analyzing multiple FF [41,42]. However, this needs to be further investigation.

Only a few reports focused on the relationship between intrafollicular VEGF level and ovarian reserve, especially in women receiving GnRH antagonist protocol IVF. Intriguingly, a report showed that there is a relative increase in superficial ovarian cortex vascularization in aging ovaries [45]. However, that study mainly focused on the whole ovary, but not a single follicle in the ovary. Cunba-Filho’s team found that VEGF concentrations in FF varied between 1020 and 1560 pg/mL in patients who underwent natural cycle IVF [46]. In contrast, the mean concentration of intrafollicular VEGF in our study in IVF patients who underwent a GnRH antagonist protocol was 1424.1 ± 146.9 pg/mL in a preovulatory leading follicle after ovarian stimulation, and this result is similar to that observed in natural cycle.

A previous study suggested that elevated VEGF concentrations in FF are connected to low oocyte quality and decreased pregnancy rate in older patients who received the long protocol [38]. In contrast, the decrease in intrafollicular and serum VEGF levels is correlated with improved ovarian response leading to increased number of retrieved oocytes and improved fertilization as well as pregnancy rates [12,47]. However, in women with polycystic ovary syndrome (PCOS), increased intrafollicular VEGF is strongly related to the happening of ovarian hyperstimulation syndrome (OHSS) [15]. Furthermore, elevated intrafollicular VEGF concentration in patients with PCOS suggests the presence of immature oocytes as well as poor fertilization rates [37,47]. In the present study, it is interesting that the rate of oocyte maturation was lower in Group A (66.3%) than that in Group B (72.2%) and Group C (75.7%) (Table 1), and a negative relationship between intrafollicular VEGF level and oocyte maturation rate was observed among different ovarian reserve groups. The findings of our study were similar to that of a previous study using GnRH Agonist Protocols [37]. Therefore, we propose that oocyte maturation is related to intrafollicular VEGF concentration regardless of IVF treatment: whether with GnRH agonist or GnRH antagonist.

In clinical practice, age, AMH, basal FSH, and AFC are often used as predictors for ovarian reserve and ovarian stimulation response. High basal FSH level, low AMH level, and reduced AFC have been associated with poor ovarian response and pregnancy outcomes [21,24,48,49]. The present study revealed significant differences in the mean values of age, serum AMH, AFC, and basal FSH among AMH groups. Ovarian response indexes, including E2 level on the hCG day and the number of retrieved oocytes, were also significantly different among the three groups (Table 1). The ovarian response indexes increased in association with AMH and AFC levels and decreased with age and basal FSH level in the study.

There was a report demonstrating that elevation of intrafollicular VEGF during ovarian stimulation in IVF treatment will increase the incidence of ovarian hyperstimulation syndrome (OHSS) [50]. For prevention of OHSS, several methods were used, which include reducing gonadotropin doses, adopting GnRH antagonist protocol, triggering with GnRH agonist or a dual trigger, and freezing all retrieved oocytes. In this study, all patients received a dual trigger (3000 U of hCG + 0.2 mg of GnRH agonist) for oocyte maturation. We found a decrease in intrafollicular VEGF concentration from Group A to Group C. There was a negative association between serum AMH level and intrafollicular VEGF concentration (Figure 1). We found that dual trigger significantly reduces intrafollicular VEGF concentration and thereby the risk of OHSS in the high AMH level group (PCOS group). Therefore, in clinical settings, intrafollicular VEGF concentration has a potential role as a predictor of OHSS occurrence. Further studies are needed to find the optimal intrafollicular VEGF cutoff for predicting the occurrence of OHSS.

Our observation (Figure 3) reveals that the production of VEGF in FF is an early event during folliculogenesis. The concentration of VEGF is increased in folliculogenesis by ovarian stimulation in IVF until follicle maturation on the hCG trigger day. It seems that lower intrafollicular VEGF concentration at the late stage of folliculogenesis is beneficial for oocyte maturation. The optimal duration of ovarian stimulation for oocyte maturation is within 10 days in the study, as indicated by the plateauing and then dropping of the VEGF secretion level in FF on stimulation days 9 and 10, respectively. Yang et al. demonstrated that the duration of ovarian stimulation has various influences on oocyte maturation in both poor and normal responders in fresh IVF cycles with GnRH antagonist treatment. Their data showed an oocyte maturation rate of 85% when the stimulation duration was up to 9 or 10 days [49]. Our result also suggests that VEGF level is related to the duration of ovarian stimulation, as well as being associated with the rate of oocyte maturation.

In this study, some of the patients in Group A were also in the group 4 (age ≥ 35 y, AFC < 5, and AMH < 1.2 ng/mL) of Patient-oriented Strategies Encompassing Individualized Oocyte Number (POSEIDON) classification and patients in POSEIDON Group 4 criteria account for 55% of low prognosis of IVF outcomes and have the poorest ovarian response, even when using a very high dose of exogenous gonadotropins [51]. Current strategies used mainly rely on evidence from poor ovarian response (POR) patients [52]. Previous studies showed that a mild stimulation protocol for POR patients is comparable, or not inferior to, conventional ovarian stimulation with high-dose gonadotropins in terms of IVF outcomes [53,54]. In our Reproductive Medicine Center, mild ovarian stimulation with a lower dose of gonadotropins was employed to treat the patients in POSEIDON Group 4 criteria. Because these cases were included in Group A, the mean total FSH dose in Group A was lower than in Group B and a little higher than in Group C. In addition, in order to prevent OHSS, we also reduced the gonadotropins dose in high-ovarian-reserve patients (Group C).

## 5. Conclusions

In conclusion, intrafollicular VEGF concentration in individual preovulatory leading follicles is negatively correlated with ovarian reserve and oocyte maturation rate and is positively correlated with serum basal FSH level in IVF patients undergoing ovarian stimulation with GnRH antagonist protocols. An elevated concentration of intrafollicular VEGF in preovulatory leading follicles is associated with low ovarian reserve, which may occur in an attempt to compensate for hypoxia. The balancing of the intrafollicular VEGF level seems to be important for the health of the follicle. More research should be conducted to elucidate the mechanisms involved.

## Figures and Tables

**Table 1:** Baseline characteristics and laboratory measurements in different ovarian reserve groups.

	Group A (AMH < 2.0 ng/mL)	Group B (2.0 ≤ AMH ≤ 6.8 ng/mL)	Group C (AMH > 6.8 ng/mL)	*p*-Value
No. of patients	81	81	23	
Age (y)	40.8 ± 3.7	36.9 ± 4.4	34.0 ± 5.1	<0.05
BMI (kg/m^2^)	21.5 ± 0.5	22.2 ± 0.6	21.7 ± 0.6	0.56
AMH (ng/mL)	0.8 ± 0.5	3.7 ± 2.5	11.0 ± 4.3	<0.05
AFC (No.)	5.3 ± 3.1	13.5 ± 5.7	25.1 ± 11.4	<0.05
Basal FSH (mIU/mL)	10.0 ± 3.9	7.2 ± 2.2	6.7 ± 2.9	<0.05
Basal LH (mIU/mL)	5.0 ± 2.2	5.2 ± 2.0	6.0 ± 3.2	0.247
Basal E2 (pg/mL)	32.8 ± 19.0	31.7 ± 20.9	37.9 ± 20.3	0.482
Basal P4 (ng/mL)	0.5 ± 0.5	0.4 ± 0.3	0.5 ± 0.4	0.929
Total FSH dose (IU)	1975.5 ± 796.6	2136.9 ± 856.4	1966.7 ± 720.1	0.514
Total LH dose (IU)	703.1 ± 529.8	757.7 ± 711.3	900.0 ± 710.8	0.632
Stimulation duration (d)	9.0 ± 1.1	9.3 ± 1.1	8.6 ± 1.0	<0.05
E2 on hCG day (pg/mL)	1039.8 ± 736.5	1793.7 ± 756.6	3569.3 ± 2133.4	<0.05
LH on hCG day (mIU/mL)	7.1 ± 6.9	2.7 ± 2.2	3.6 ± 2.9	<0.05
P4 on hCG day (ng/mL)	0.7 ± 0.6	1.2 ± 1.9	1.2 ± 0.8	0.17
VEGF in FF (pg/mL)	1692.2 ± 124.1	1222.4 ± 137.8	627.9 ± 66.7	<0.05
Oocyte No.	3.7 ± 3.6	12.0 ± 7.3	23.7 ± 12.0	<0.05
MII oocyte No.	3.3 ± 0.6	7.7 ± 0.9	18.3 ± 1.9	<0.05
Oocyte maturation rate *	264/398 (66.3%)	694/961 (72.2%)	499/659 (75.7%)	<0.05

* Oocyte maturation rate = (MII oocyte No./total oocyte retrieved No.) × 100%. The data were expressed as the mean ± standard deviation (SD). Abbreviations: AFC, antral follicle count; AMH, anti-Müllerian hormone; E2, estradiol; FSH, follicle-stimulating hormone; hCG, human chorionic gonadotropin; LH, luteinizing hormone; P4, progesterone; VEGF, vascular endothelial growth factor; FF, follicular fluid; MII, metaphase II.

## Data Availability

The data presented in this study are available upon request from the corresponding author. The data are not publicly available due to patient privacy restrictions.
